# Optogenetic and Chemical Induction Systems for Regulation of Transgene Expression in Plants: Use in Basic and Applied Research

**DOI:** 10.3390/ijms23031737

**Published:** 2022-02-03

**Authors:** Evgeniya S. Omelina, Anastasiya A. Yushkova, Daria M. Motorina, Grigorii A. Volegov, Elena N. Kozhevnikova, Alexey V. Pindyurin

**Affiliations:** 1 Department of Regulation of Genetic Processes, Institute of Molecular and Cellular Biology, SB RAS, 630090 Novosibirsk, Russia; omelina@mcb.nsc.ru (E.S.O.); a.yushkova@g.nsu.ru (A.A.Y.); d.motorina@g.nsu.ru (D.M.M.); g.volegov@g.nsu.ru (G.A.V.); 2 Laboratory of Biotechnology, Novosibirsk State Agrarian University, 630039 Novosibirsk, Russia; e_zeste@yahoo.com; 3 Faculty of Natural Sciences, Novosibirsk State University, Novosibirsk 630090, Russia; 4 Laboratory of Experimental Models of Cognitive and Emotional Disorders, Scientific-Research Institute of Neurosciences and Medicine, 630117 Novosibirsk, Russia

**Keywords:** optogenetics, chemical induction, transgene expression regulation, plants, agriculture

## Abstract

Continuous and ubiquitous expression of foreign genes sometimes results in harmful effects on the growth, development and metabolic activities of plants. Tissue-specific promoters help to overcome this disadvantage, but do not allow one to precisely control transgene expression over time. Thus, inducible transgene expression systems have obvious benefits. In plants, transcriptional regulation is usually driven by chemical agents under the control of chemically-inducible promoters. These systems are diverse, but usually contain two elements, the chimeric transcription factor and the reporter gene. The commonly used chemically-induced expression systems are tetracycline-, steroid-, insecticide-, copper-, and ethanol-regulated. Unlike chemical-inducible systems, optogenetic tools enable spatiotemporal, quantitative and reversible control over transgene expression with light, overcoming limitations of chemically-inducible systems. This review updates and summarizes optogenetic and chemical induction methods of transgene expression used in basic plant research and discusses their potential in field applications.

## 1. Introduction

Besides various basic research needs, transgenic plant engineering is aimed, first of all, at the introduction of new traits that increase resistance to abiotic and biotic stresses. Abiotic stress includes adverse environmental conditions, such as drought, salinity, extreme temperatures and pollutants. For example, drought causes a drastic effect on yield in plants; thus, development of drought-tolerant plants is among the priority tasks of applied research in agriculture. It is known that drought tolerance is a complex polygenic trait controlled by a large number of genes [1]. Several different transgenes were found to increase drought and salt tolerance in such important food crops as *Glycine max* (soybean) [2,3,4,5,6,7] and *Zea mays* (maize) [8,9,10]. Soybean provides an essential protein source to the human diet. Maize is used as food and fodder and has commercial value in ethanol production. Unlike soybean and maize, engineering of transgenic wheat *Triticum aestivum*, which is one of the most important cereal and staple food crops, has limitations due to its complex and large hexaploid genome [11]. However, some progress in the development of transgenic wheat with drought tolerance was also reported [12,13,14,15,16,17,18]. In addition to food crops, cotton *Gossypium hirsutum,* which is one of the most important natural sources of fiber, was also genetically modified to enhance tolerance to drought and salinity [19,20]. There are many food crops tolerant to biotic stress, e.g., different insect pests. Nowadays, more than 700 *cry* toxin genes from *Bacillus thuringiensis* (Bt) with high specificity towards different insect taxa are known [21]. A lot of Bt-plants were engineered and commercialized (e.g., maize, cotton, potato, rice). As a result, currently, in the USA, Bt-maize and Bt-cotton represent 82% and 88% of total maize and cotton production, respectively [22]. There are several other transgenic crops with different useful traits. For instance, sunflower *Helianthus annuus* with a resistance to a fungal pathogen *Alternaria helianthi* [23], transgenic radish *Raphanus sativus* with heat tolerance [24], transgenic flax *Linum usitatissimum* with an increased production of medically beneficial glycosyltransferases [25] and many others. Thus, the introduction of different transgenes in plants helps to overcome a plethora of challenges in agriculture by enhancing resistance to abiotic and biotic stresses. However, there is a trade-off between plant growth yield and tolerance to stresses [26], since transgene expression may result in defense-associated fitness costs [27,28].

Increased tolerance to a severe stress without any yield penalty is a focal point of today’s plant genetic engineering. One of the simplest solutions is to use tissue-specific promoters, as tissue- and temporally-controlled transgene expression should minimize the adverse effects on plant physiology. The characterization of some tissue-specific plant promoters allows for developing synthetic regulatory elements that drive state- and tissue-specific transgene expression [29,30,31]. However, such promoters typically activate transgene expression limited to one or a few tissues or cell types, which may be unsuitable for many applications. Moreover, tissue-specific promoters do not allow temporal or quantitative regulation of gene expression. Besides that, plants have endogenous promoters which are induced under certain stress conditions, e.g., salt-, low/high temperature-, drought-, and pathogen-inducible promoters are known [32]. However, such promoters also cannot ensure precise spatiotemporal control of transgene expression, especially in complex environmental conditions. Thus, inducible systems providing high spatiotemporal resolution of target gene expression are useful instruments in plant molecular biology and biotechnology. A successfully constructed inducible gene expression system should be highly specific to the inducer as well as having a high dynamic range of response to its concentrations, fast response upon induction and a rapid switch-off. Compared with constitutive and tissue-specific gene expression systems, inducible systems have the following advantages:

- without specific induction, the system is off and the gene of interest is not expressed;
- upon induction, the promoter is activated, enabling the target gene transcription in specific plant growth periods and tissues;
- the induction can be switched-off if it is needed [33].

Inducible expression systems can be activated by different factors, e.g., chemicals and light. In a typical chemical induction system, expression of two genes is controlled by suitable promoters and one of the genes encodes the transcription factor (activator or inhibitor), which can specifically bind to the promoter of the second gene (target or reporter gene). The transcription factor encoded by the first gene may be artificially constructed (chimeric transcription factor) and its promoter may be ubiquitous or tissue-specific to obtain strong and universal or local expression, respectively. The promoter of the reporter gene contains the binding sites of the transcription factor and should have a low level of basal expression to prevent the leakage of the system without induction. In plants, transgenes can be induced by various chemically-inducible systems based on activation, inactivation, and de-inhibition [34]. Commonly used chemical expression systems are tetracycline-, steroid-, insecticide-, copper-, and ethanol-induced. Importantly, a chemical inducer should not be a plant metabolite and should have no toxic effect on plant viability. Chemical inducers can be applied to soil, plant surfaces, or plant growth media. Currently there is a list of officially registered agrochemicals that can be used as inducers (https://www.eppo.int/ACTIVITIES/plant_protection_products/registered_products (accessed on 21 November 2021)). 

**Figure 1:**
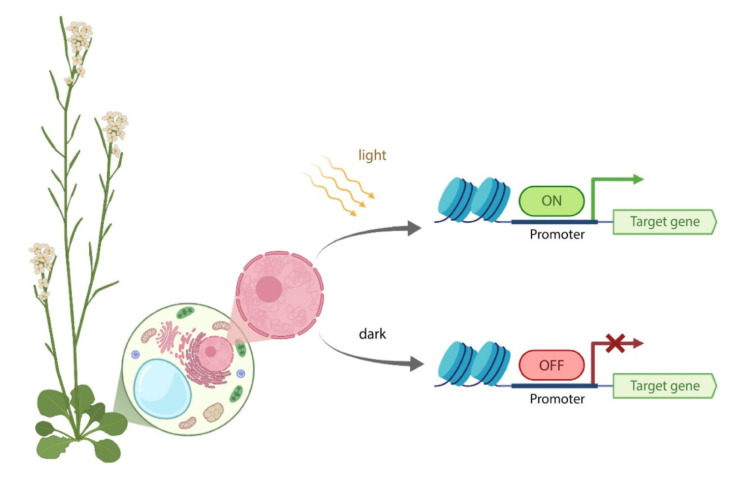
General strategy for optogenetic systems in plants. In dark, the system is turned off, the target gene does not express. Upon illumination with the light of a certain wavelength, the optogenetic system is activated, resulting in the transcription of the target gene.

Unlike chemical induction systems, regulation of gene expression in plants using optogenetic systems (Figure 1) has recently started to develop. In comparison with the chemical induction systems, optogenetic tools have some advantages. Firstly, light can be focused on a small plant area or even within a plant cell. Secondly, induction with light enables one to control the intensity and duration of exposure. Thirdly, light allows one to instantly turn on or off gene expression following the light switch. Moreover, as opposed to chemical inducers, light does not depend on the diffusion rate and exhibits far fewer off-target effects. Despite these facts, optogenetic systems have a disadvantage, which limits their usage in plants. Specifically, plants are phototrophic organisms and require exposure to sunlight, and the light of different wavelengths and intensities required for plant growth [35] may non-specifically activate optogenetic systems [36].

Over the years, the technology for developing both chemical- and light-inducible systems has evolved and become an invaluable tool for modern agricultural research. In this review, we describe optogenetic and chemical gene regulation systems used in plants and evaluate their benefits and pitfalls.

## 2. Optogenetic and Chemical Induction Systems

### 2.1. Optogenetics and Photoreceptors

Photoreceptors used in the design of optogenetic tools can be divided into several classes according to their chromophores and light-sensing protein domains: UV- and blue-light-sensing flavoproteins, e.g., photoreceptors with light-oxygen-voltage (LOV) sensing domains; cryptochromes incorporating flavin adenine dinucleotide (FAD) as a chromophore; blue-light-utilizing FAD (BLUF) domains; bacterial channelrhodopsins, sensing blue, green, and yellow light and incorporating retinal as a chromophore; far-red and near-infrared (NIR) light-sensing phytochromes utilizing various tetrapyrroles, the products of enzymatic degradation of a heme, as chromophores.

**Figure 2:**
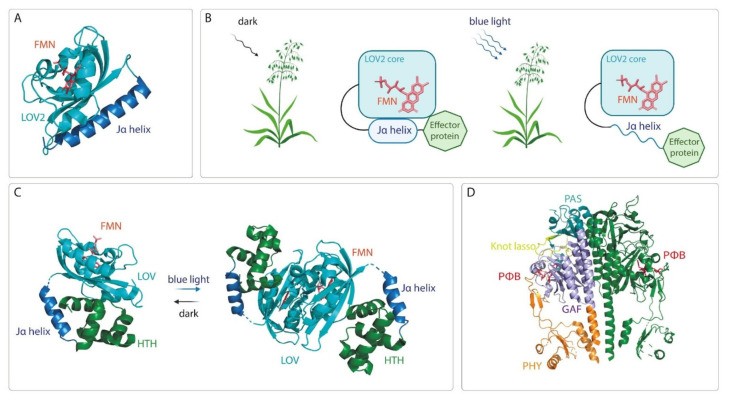
Domain structure and conformational changes of photoreceptors upon absorption of light. (**A**) Shown is the crystal structure of the LOV2 domain of *A*. *sativa* phototropin 1 (PDB ID 2V0U). The LOV2 domain is presented as ribbons (cyan) and the flavin chromophore as a stick model (red). (**B**) Schematic structure of the LOV2 domain from *A*. *sativa* fused with effector protein in darkness and under blue light. In dark, Jα helix docks onto the LOV2 core; under blue light it undocks, making the effector protein available for protein-protein interaction. (**C**) Homodimerization of the LOV domain containing protein EL222 from *E*. *litoralis* under blue light. The protein structure is presented as ribbons (cyan, blue, green) and the flavin chromophore is shown as a stick model (red). The crystal structure of the LOV domain from *E*. *litoralis* (PDB ID 3P7N) was used for visualization. HTH – DNA binding helix–turn–helix domain. (**D**) Typical domain structure of the photosensory module of the plant phytochromes. The PAS, GAF, and PHY domains are shown in cyan, purple, and orange ribbons, respectively; the C-terminal output module is not shown. The chromophore (PФB) is shown as a stick model (red). The crystal structure of *A*. *thaliana* PhyB photosensory module (PDB ID 4OUR) was used for visualization.

The most popular optogenetic systems for activation and suppression of gene expression are constructed using the LOV domain proteins, cryptochromes and phytochromes. LOV domains belong to the Per-Arnt-Sim (PAS) protein family [37]. The small size (about 11–15 kDa) and the presence of flavin chromophores in most, if not all, plant cell types are key advantages of these domains as optogenetic tools. The LOV2-Jα photoswitch from *Avena sativa* phototropin 1 can undergo light-induced conformational changes [38]. The LOV2 domain contains an amphipathic Jα helix C-terminal to the LOV2 core which docks onto the β-scaffold of the core in darkness and undocks from the β-scaffold under blue light (Figure 2A,B) [39]. This ability of the LOV2 domain-containing proteins was used to engineer several blue light-induced cellular regulators by fusing Jα helix to a protein of interest (Figure 2B) [40,41,42,43,44,45,46].

One more strategy to develop the LOV-derived optogenetic tools is based on light-induced homodimerization. This approach utilizes the LOV domain-containing protein EL222 from the marine bacterium *Erythrobacter litoralis* HTCC2594, which binds the flavin mononucleotide (FMN) chromophore [47]. The structure of EL222 is classified into three parts: an N-terminal LOV domain, the connector Jα-helix and a C-terminal helix–turn–helix (HTH) DNA-binding domain (Figure 2C). In darkness, the LOV domain binds the HTH domain, preventing EL222 from dimerization and DNA binding [47]. Blue light triggers the photochemical formation of a protein/flavin adduct within the LOV domain, disrupting inhibitory LOV/HTH interactions and allowing EL222 to form dimers and bind DNA [48,49]. 

Besides systems based on the LOV domain proteins, optogenetic tools are often based on cryptochromes. Cryptochromes are found in all kingdoms of life. In plants, they regulate growth and development. Cryptochromes have a chromophore-binding photolyase homology region (PHR) and incorporate FAD [50,51]. Blue light induces an intramolecular redox reaction, which involves the FAD molecule and conserved tryptophan amino acid residues in a protein backbone, and results in structural changes and signal transmission. To develop optogenetic tools, blue light-induced heterodimerization of cryptochrome 2 (CRY2) with the cryptochrome-interacting basic helix–loop–helix 1 (CIB1) protein from *Arabidopsis thaliana* is often used [52,53].

**Figure 3:**
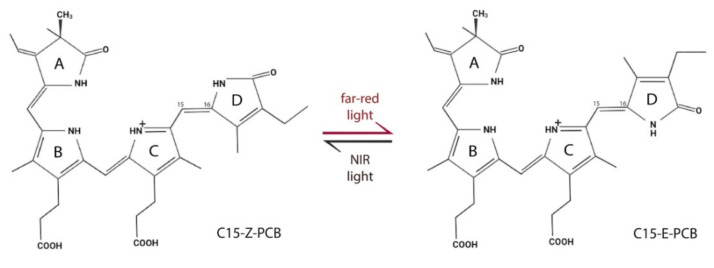
Light-driven changes in structure of tetrapyrrole chromophore PCB. Reversible Z/E isomerization of the C15/C16 double bond in bound PCB chromophore under illumination with far-red (640–680 nm) and NIR light (740–780 nm).

Unlike the LOV domain containing proteins and cryptochromes regulated by blue light, optogenetic systems based on the usage of phytochromes are controlled by long wavelength light. Phytochromes are photoreceptors found in plants, bacteria, cyanobacteria, and fungi, playing essential roles in light-adaptive processes. Phytochromes share common domains in a photosensory core module (PCM), consisting of PAS, GAF (cGMP phosphodiesterase/adenylate cyclase/FhlA), and PHY (phytochrome-specific) domains (Figure 2D). Specifically, bacterial and fungal phytochromes incorporate tetrapyrrole chromophore biliverdin IXα (BV). In contrast, in plants and cyanobacteria, BV is enzymatically reduced to phytochromobilin or phycocyanobilin (PCB), which bind to plant and cyanobacterial phytochromes, respectively. In all phytochromes, the respective tetrapyrrole chromophore is covalently attached via the C3 side chain of the tetrapyrrole A-ring to a conserved cysteine in the PAS or GAF domain. Photocycle includes reversible photoisomerization of the chromophore around its 15/16 double bond (Figure 3). This causes rotation of a D-ring and conformational changes in the protein backbone, which are transferred to an output module, also known as effector domain. The output module is typically represented by a histidine kinase. However, other effectors have also been reported, e.g., domains that interact with DNA repressors [54], and diguanylate cyclase or phosphodiesterase domains involved in second messenger signaling [55]. To develop optogenetic systems, plant and bacterial phytochromes are currently used. The plant phytochromes PhyA and PhyB from *A*. *thaliana* are readily activated by far-red (640-680 nm) light and rapidly deactivated by NIR (740-780 nm) light. In the active state, PhyA and PhyB interact with phytochrome-interacting factors 3 (PIF3) or 6 (PIF6) [56,57]. Interaction of PhyA and PhyB with PIF3 was exploited to develop a light-switchable gene expression system [58,59]. 

### 2.2. Optogenetic Systems in Plants

**Figure 4:**
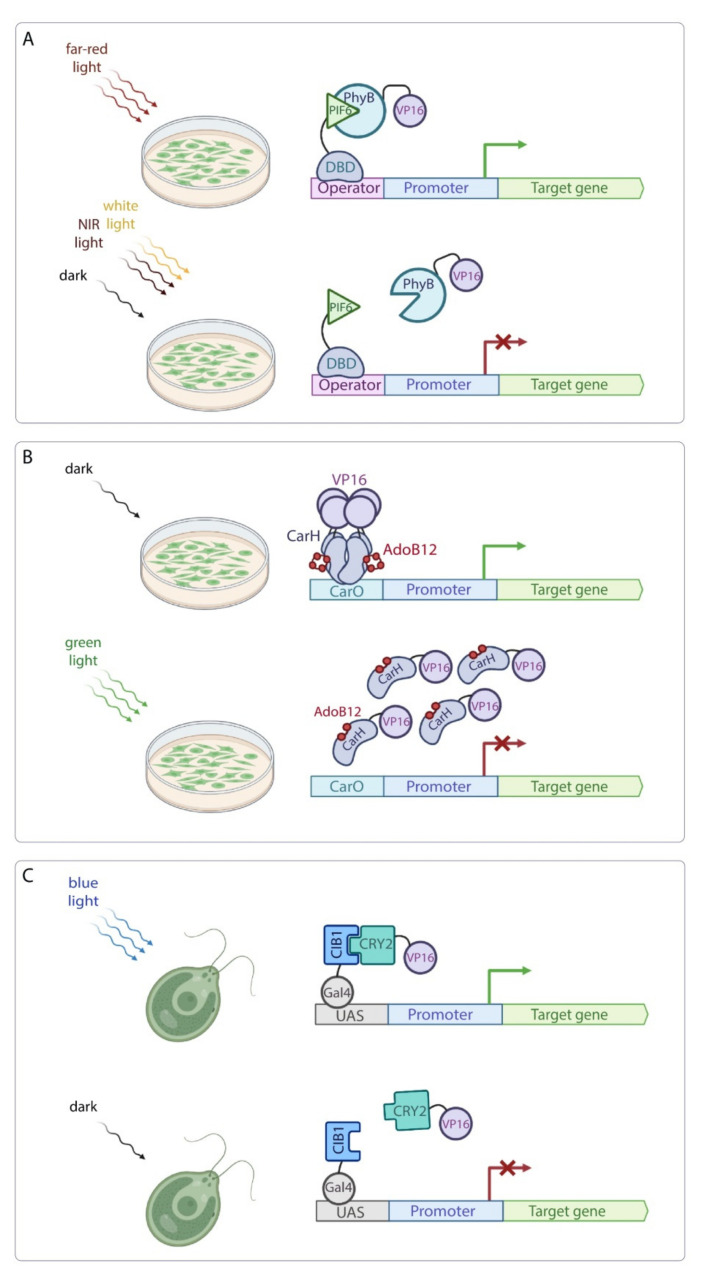
Application of optogenetic tools to control transgene expression in plants. (**A**) Application of the PhyB-PIF6 optogenetic pair in plant protoplasts. Far-red light (640–680 nm) induces heterodimerization of the PhyB and PIF6, resulting in activation of the reporter gene transcription. Incubation in darkness or illumination with NIR light (740–780 nm) inhibits this process. DBD – DNA-binding domain of the TetR, E or PiP proteins. (**B**) Green light-induced transcription inhibition in plants. In darkness, bacterial photoreceptor CarH fused to the VP16 AD and linked to the chromophore AdoB12 binds to the CarO operator resulting in transcription activation of the target gene. Illumination with 525 nm light induces dissociation of the CarH tetramers releasing CarO and subsequent inhibition of the reporter gene expression. (**C**) Blue light-induced gene expression system in alga *C. reinhardtii*. Blue light (460–480 nm) stimulates CIB1 and CRY2 heterodimerization and thus brings the VP16 AD closer to Gal4 DBD that results in the activation of the target gene transcription. In darkness, CRY2-VP16 complex dissociates from CIB1-Gal4 DBD. UAS—upstream activation sequence; Gal4—Gal4 DBD.

In plants, the following optogenetic systems are used to regulate transgene expression. A split system based on far-red light-dependent interaction of PhyB and PIF6 from *A. thaliana* is used in *Nicotiana benthamiana* and *A. thaliana* protoplasts to induce reporter gene transcription under far-red light (660 nm). PIF6 (amino acids 1–100) is fused to a DNA-binding domain (DBD) of the tetracycline repressor protein (TetR), the macrolide repressor protein (E) or the pristinamycin repressor protein (PiP), which bind their operator sites in the reporter constructs upstream of a minimal promoter and the gene of interest. The N-terminus of PhyB is fused to a nuclear localization signal (NLS) and the viral VP16 activation domain (AD) [60]. In the 660 nm-illuminated samples the reporter gene reveals high expression levels, while expression in the dark-incubated protoplasts remains at basal levels (Figure 4A). To prevent the system’s activation under white light, 740 nm light of increasing intensities was used, resulting in a complete repression of the transgene comparable to the dark-incubated samples. The DBD of the E protein showed higher luciferase reporter levels compared to the TetR and PiP DBDs as well as higher induction ratio of the active state to the repressed state.

In addition to transcription activating systems, a green light-induced inhibition system to control transgene expression in plant cells was developed recently [61]. Green light (525 nm) is an attractive wavelength for optogenetic control in plants as plant photoreceptors show reduced activity in this segment of the light spectrum [62]. Additionally, green light is effective in stimulating photoreceptor function in plants [63]. This system was engineered using the bacterial photoreceptor CarH. CarH is a light-responsive transcription factor which regulates the expression of a carotenogenic gene cluster and uses the coenzyme, 5′deoxyadenosylcobalamin (AdoB12), as a chromophore. In darkness, CarH linked to the AdoB12 chromophore binds to the DNA operator CarO as a tetramer. Under green light, photolysis disrupts the Co−C bond in AdoB12 leading to the disassembly and release of the CarH tetramer from CarO operator [64]. This system was tested in *A*. *thaliana* protoplasts [61,65]. CarH fused to the VP16 AD and linked to AdoB12 binds to the CarO sequence resulting in transcriptional activation of the reporter gene in the dark. Upon green light illumination, CarH-VP16 tetramers dissociate and release CarO, which becomes unable to activate reporter gene expression (Figure 4B). However, this system has limitations for in vivo applications, as the AdoB12 chromophore is not produced in plants and should be added exogenously.

The gene expression system based on the blue light-controlled heterodimerization strategy was developed to engineer the blue light-inducible hydrogen-producing transgenic alga *Chlamydomonas reinhardtii* [66]. The CRY2 and CIB1 proteins from *Arabidopsis* were fused with the viral VP16 AD and the yeast Gal4 DBD, respectively. Under blue light (460-480 nm), CRY2 and CIB1 form heterodimers, resulting in the reporter gene transcription activation (Figure 4C). The artificial miRNA (amiR-D1) targeting the photosystem II reaction-center protein D1 (encoded by the *psbA* gene) was expressed under the control of yeast upstream activation sequence (UAS). Upon blue light illumination, the transcription level of amiR-D1 was increased and its target gene *psbA* was down-regulated in transgenic alga that resulted in the improved hydrogen yield under blue light [66].

**Figure 5:**
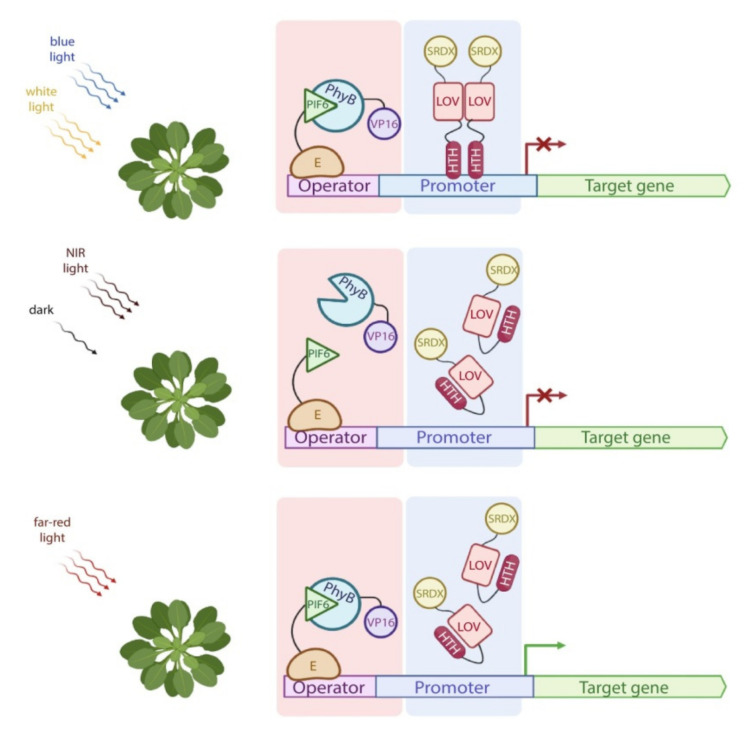
Control of transgene expression in plants using the PULSE system. Upon illumination with blue (460–480 nm) or white light, PhyB interacts with PIF6. However, transcription of the reporter gene is blocked by blue-off module, which is based on the blue light-induced homodimerization of the EL222 LOV domain fused to a plant transcriptional repressor domain SRDX. Under NIR light (740–780 nm) or in darkness, PhyB does not interact with PIF6 and blue-off module is not active. Under far-red illumination (660 nm), blue-off module is also not active, while PhyB and PIF6 form heterodimers, resulting in the transcription activation of the reporter gene. E—DBD of the macrolide repressor protein E. Pink and blue boxes are the far-red light activation and blue light inhibition (blue-off) modules, respectively.

Plant-usable light-switch elements (PULSE) is a dual-controlled and insensitive to ambient light optogenetic system for activating transcription of transgenes based on PhyB and PIF6 proteins [67]. It consists of two components: a far-red light-inducible module for activation of the reporter gene transcription and a blue light-regulated module ensuring effective repression of the reporter gene (Figure 5). 

A far-red light activation module contains constitutively expressed PhyB–VP16 and the chimeric protein E–PIF6, consisting of the macrolide repressor protein from bacteria *Escherichia coli* (E) [60] fused to the first 100 amino acids of PIF6. Under 640–680 nm light, PhyB and PIF6 form heterodimers resulting in activation of the reporter gene transcription. The blue-off module is engineered from the LOV-based photoreceptor EL222 from *E*. *litoralis* [47], which is fused to the plant ERF-associated amphiphilic repression domain SRDX [68]. EL222 dimerizes and binds to its target DNA sequence under blue light. Thus, PULSE enables effective control of transgene expression under standard plant growth conditions as transcription is repressed under white light by the blue-off module and is activated only under far-red light. This system was tested in both the protoplasts and whole *Arabidopsis* transgenic plants and in *N. benthamiana* leaves; it seems to be very promising for a variety of applications, including light-dependent control of plant immunity. However, it should be noted that PhyB is a plant phytochrome which can interact with endogenous proteins and potentially affect endogenous metabolic pathways.

In plants, optogenetic tools can be also used for manipulations with stomatal kinetics. For that, the synthetic blue light-induced K+ channel 1 (BLINK1) was expressed in guard cells surrounding stomatal pores in *A*. *thaliana* [69]. BLINK1 was engineered by fusing the LOV2-Jα photosensory module (Figure 2A) to various regions of the small viral K+ channel pore Kcv known to be mechanically important for channel gating [70]. That results in the generation of a blue light-inducible K+ channel. It was shown that BLINK1 accelerates stomatal kinetics, leading to an increase in the speed of stomatal opening under blue light and closing in darkness. Additionally, BLINK1 improves water use efficiency without penalty in carbon fixation. As a result, the plants with the BLINK1 transgene drive a 2.2-fold increase in biomass compared to wild-type counterparts [70]. 

Optogenetic systems are also applied to regulate plant growth. For instance, for synthesis of chromophore retinal from β-carotene, the green-light-gated anion channelrhodopsin ACR1 from the green alga *Guillardia theta* and marine bacterial β-carotene 15,15′-dioxygenase were used in transgenic tobacco plants. This allowed for controlling plant growth and leaf development [71]. ACR1 was also used to control the aperture of plant stomata [72]. Continuous green light illumination leads to the loss of turgor of guard cells expressing ACR1 resulting in closure of stomata in conditions provoking stomatal opening in wild type. Another two anion channelrhodopsins, the blue light-controlled ACR2 from *G. theta* [73] and the green light-controlled ZipACR from *Proteomonas sulcata* [74], were used in *N. benthamiana* mesophyll cells and leaves and *Nicotiana tabacum* pollen tubes to study plant ion and electrical signaling [75]. Additionally, the mutant variant of the channelrhodopsin-2 protein (channelrhodopsin-2-XXL) from the green alga *C. reinhardtii* [76] was used to study plant electrical signaling in *Arabidopsis* leaf cells [77]. 

### 2.3. Chemical Induction Systems in Plants

In plants, chemical induction systems are used for activation or repression of the transgene expression. In this chapter, we focus primarily on the research aimed at improving agricultural performance of plants by chemically-inducible regulation of transgene expression. For this purpose, the tetracycline-, the steroid-, the copper- and the ethanol-regulated systems are currently used.

**Figure 6:**
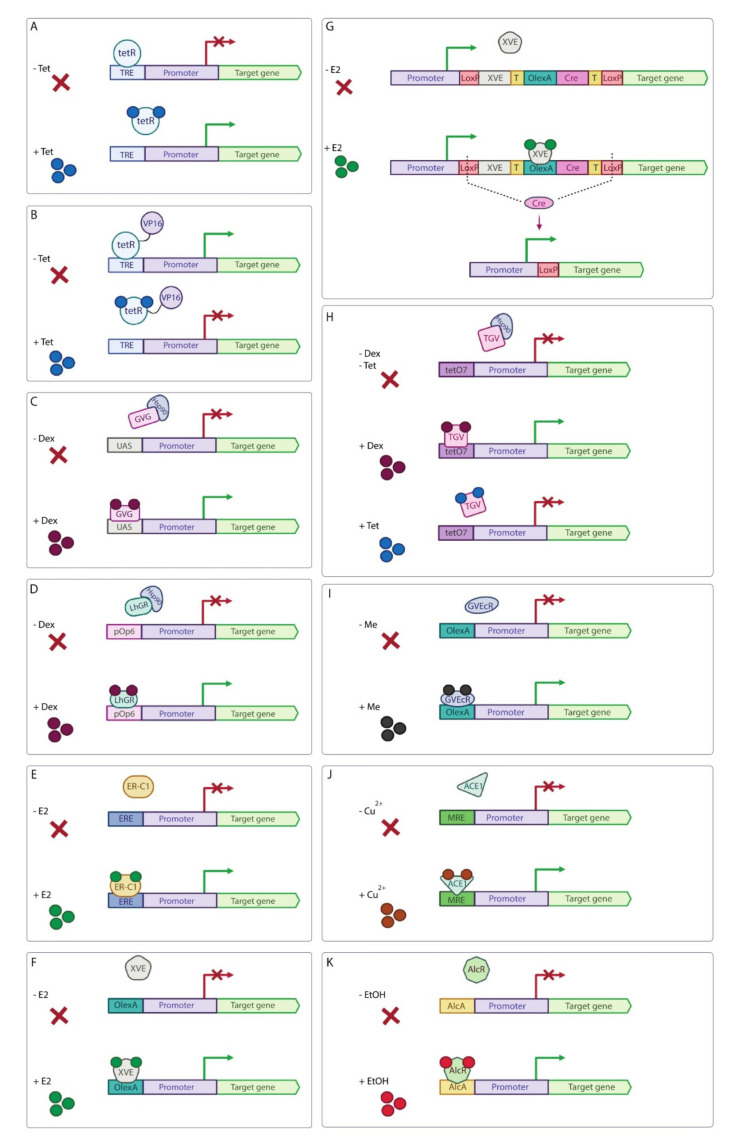
Schematic representation of chemically-inducible systems for regulation of transgene expression in plants. (**A**) The Tet-derepressible system. In the absence of tetracycline (Tet), tetR binds to TRE sequences, and the target gene is not transcribed. Upon Tet addition, tetR cannot bind to TRE, allowing the target gene transcription. (**B**) The Tet-off system. In the absence of Tet, tetR-VP16 binds to TRE resulting in activation of the target gene. In the presence of Tet, tetR-VP16 cannot bind to TRE to induce the transgene expression. (**C**) The GVG/UAS system. In the absence of dexamethasone (Dex), GVG binds to the regulatory protein Hsp90 forming an inactive complex. Dex addition results in the dissociation of GVG from the Hsp90 protein and its binding to UAS upstream of the target gene. (**D**) The pOp6/LhGR system. In the absence of Dex, LhGR binds to the regulatory protein Hsp90, and, thus, is inactive. In the presence of Dex, LhGR binds to pOp6 sequence to activate the transgene expression. (**E**) ER-C1 system. In the absence of β-estradiol (E2), ER-C1 does not bind to ER elements (ERE). Once activated by E2, ER-C1 binds to ERE and activates target gene expression. (**F**) The XVE system. In the absence of E2, XVE does not bind to the LexA operator sequence (OlexA). After treatment with E2, XVE binds to OlexA and activates the target gene expression. (**G**) The XVE-controlled Cre/loxP system. The XVE and Cre expression cassettes are located between the constitutive promoter and the target gene flanked by two loxP sites. In uninduced condition, the target gene is not expressed. In the presence of inducer (E2), XVE activates the Cre transcription, resulting in the fusion of the constitutive promoter and the target gene due to Cre/loxP-mediated recombination. T—transcription terminator. (**H**) A dual-controlled TGV system. In the absence of Dex and Tet, TGV binds to the Hsp90 protein, forming an inactive complex. Upon Dex addition, TGV separates from Hsp90, binds to the Tet operator sequence (tetO7) and activates the target gene expression. In the presence of Tet, the TGV protein dissociates from the reporter gene promoter and the level of the target gene transcription drops to uninduced levels. (**I**) The GVEcR system. Without methoxyfenozide (Me), GVEcR does not bind to the LexA binding site (OlexA) and the target gene is not transcribed. In the presence of Me, GVEcR binds to OlexA and activates the target gene transcription. (**J**) The copper-inducible system. In the absence of copper (Cu^2+^), the transcription factor ACE1 does not bind to metal responsive element (MRE) and the transgene is not transcribed. In the presence of copper ions, ACE1 binds to MRE and activates the target gene transcription. (**K**) The ethanol-inducible system. Without ethanol (EtOH), the transcription factor AlcR does not bind to the promoter of the *AlcA* gene (AlcA) and the transgene is not transcribed. After addition of ethanol, AlcR binds to AlcA and activates the transgene expression.

The tetracycline (Tet)-activated system was originally adapted for use in plants quite a long time ago. Tet-dependent promoters are developed by placing a Tet response element (TRE) consisting of the tetracycline operator (tetO) sequence repeats upstream of a minimal promoter. Two types of Tet-inducible systems were applied in plants: the Tet-derepressible system based on the original tetR repressor protein and the Tet-off system based on the chimeric protein tetR-VP16. In the Tet-derepressible system in the absence of tetracycline, the tetR repressor binds TRE sequences, preventing the reporter gene promoter from transcription initiation [78]. Upon Tet addition, the tetR protein binds the Tet molecule and separates from TRE, thus, releasing the reporter gene expression (Figure 6A). In the Tet-off system, TRE is recognized by the tetR protein fused to the VP16 AD (Figure 6B) [79]. In the absence of Tet, the chimeric tetR-VP16 protein binds TRE, activating the reporter gene transcription [78]. Upon Tet addition, the tetR-VP16 protein cannot bind to TRE, thus, the reporter gene is not transcribed. In the Tet-inducible systems, expression of the reporter genes can be regulated by Tet and its derivatives, e.g., doxycycline [80].

The Tet-derepressible system was used for expression of the *β-glucuronidase* gene in tobacco [81]. In this system, tetR was expressed under the control of the strong and ubiquitous CaMV35S promoter from the plant pathogen cauliflower mosaic virus. In the absence of Tet, there was no expression of the *β-glucuronidase* gene, but it was induced after Tet treatment. This system was also used to control expression of the β-glucuronidase and GFP proteins in a tobacco Bright Yellow 2 (BY2) cell line [82], as well as the “wild-type” CDC2aAt and mutant CDC2aAt.N146 proteins in tobacco protoplast cultures [79]. The Tet-off system was used to inhibit expression of the GFP protein in *A. thaliana* [83] and the β-glucuronidase protein in the moss *Physcomitrella patens* [84]. Moreover, to avoid the high-level expression of the tetR and the usage of Tet in non-inducing conditions, the Tet-off system was used in combination with a heat-shock system in tobacco [85]. The main advantages of the Tet-inducible systems are the low background expression level and the small amount of Tet to launch the system. However, the major limitation of these systems for use in field experiments is the nature of Tet. Since it is an antibiotic, its usage in applied research is strongly not recommended.

The most common strategy to apply a steroid-inducible expression system in plants is fusion of a DBD with an AD and the animal steroid nuclear receptor regulatory domain, e.g., glucocorticoid receptor (GR) or estrogen receptor (ER). The GVG/UAS and pOp6/LhGR systems for the regulation of transgene expression in plants are based on rat GR. GVG is a steroid-induced chimeric transcription factor consisting of the yeast Gal4 DBD, the VP16 AD and the rat GR ligand binding domain (LBD) [86]. In the absence of steroid ligands (e.g., dexamethasone), GVG via GR LBD interacts with the regulatory heat shock protein Hsp90, forming an inactive complex. Upon steroid addition, the binding of steroids to the GR LBD results in the dissociation of GVG from the Hsp90 regulatory protein, allowing GVG to bind the UAS and activate expression of the reporter gene (Figure 6C). The GVG/UAS system was applied for regulation of expression of the *luciferase* reporter gene using dexamethasone in transgenic tobacco and *Arabidopsis* plants [86] and for development of the inducible high-level mRNA amplification system for the human *gamma interferon* gene in transgenic tobacco [87]. The GVG system was also tested in the legume *Lotus japonicas* to express the *β-glucuronidase* gene, as well as sense and antisense RNA for transcription factors NDX and CPP1 to study their functions. However, there is also a report that this system is not suited for use in legumes, since it itself caused growth disturbances in transgenic plants [88]. 

The pOp6/LhGR system is based on the chimeric LhGR protein consisting of bacterial high affinity DNA binding mutant lacI His17 DBD, the yeast Gal4 AD and the rat GR LBD. The pOp6 promoter is a chimeric sequence containing six bacterial lac operons upstream of a minimal CaMV35S promoter from the plant pathogen cauliflower mosaic virus (Figure 6D). This system provides a highly sensitive chemically-inducible transgenic expression tool for tobacco; it may be suitable for many other plants [89]. The pOp6/LhGR system was recently combined with tissue- or cell type-specific promoters to provide a toolbox for inducible, cell type-specific expression in *Arabidopsis* [90]. As a result, 19 well-characterized and stable driver lines expressing the LhGR transcription factor under the control of tissue-specific promoters in most cell types in *Arabidopsis* were developed with a focus on the root apical meristem, the shoot apical meristem, and the cambium. This system was also applied in transgenic tobacco to regulate expression of the *β-glucuronidase* gene and the cytokinin-biosynthetic gene *ipt* [91]. It was shown that the GR LBD is sufficient for practically complete inhibition of the LhGR activity in the absence of dexamethasone. Dexamethasone-inducible pOp6/LhGR was also adjusted for application in monocotyledonous species (rice) [92]. To achieve that, the LhGR transcription factor was codon-optimized and used to induce expression of β-glucuronidase in transgenic plants. However, severe developmental abnormalities were observed in transgenic monocotyledonous plants grown on high concentrations of dexamethasone. At the same time, its low levels were sufficient to induce the reporter gene expression and not inhibit the transgenic rice seedlings growth [92]. In another study, the LhGR2 transcription factor with codon-optimized Gal4 AD sequence [93] under the control of adjusted maize ubiquitin promoter and the pOpIn2 bidirectional reporter cassette, which is non-toxic for monocots [94], were used to induce the *β-glucuronidase* gene expression in transgenic rice *Oryza sativa* spp. *japonica* [95]. Dexamethasone-induced reporter gene activity was dose- and time-dependent. Additionally, the induction of transgene expression with another glucocorticoid derivative, triamcinolone acetonide (TA), was comparable, if not higher, than with dexamethasone. The system was functional at later stages of plant development in soil-grown plants; long-term induction has no negative effects on the development and growth of rice [95].

β-estradiol (E2) induction system is based on human ER and contains the chimeric ER-C1 or XVE proteins. ER-C1 is the fusion of the AD of corn activator C1 and the human ER, while the promoter of the reporter gene consists of ER elements (ERE) and minimal CaMV35S promoter (Figure 6E). The E2 induction system based on ER-C1 was used in maize BMS (Black Mexican Sweet) cell line to regulate expression of the C1/R and P transcription factors responsible for activating flavonoid synthesis and the *luciferase* reporter genes by the addition of E2 [96].

E2- and dexamethasone-inducible systems have been actively used in recent years to study plant defense mechanisms for restricting pathogen attack. Upon recognition of the effector proteins secreted by pathogens, plants can activate an innate immune system that includes pattern-triggered immunity (PTI) and effector-triggered immunity (ETI). PTI is activated by cell-surface pattern-recognition receptors, whereas ETI is triggered by intracellular nucleotide-binding domain, leucine-rich-repeat containing receptors (NLRs). One of the current challenges in understanding these immune pathways is to activate intracellular NLRs without inducing PTI. To achieve that, the bacterial effector proteins AvrRps4 and AvrRpt2 were expressed in *Arabidopsis* with E2 system [97] and dexamethasone system [98], respectively. Thus, these chemically-inducible systems allow for the study of ETI in the absence of PTI [97,98].

The chimeric protein XVE consists of the DBD from the bacterial LexA protein, the VP16 AD and the human ER LBD. The corresponding promoter of the target gene consists of the eight copies of the LexA operator sequence (OlexA) and minimal CaMV35S promoter (Figure 6F). Since the XVE system has a very low level of basal activation and since E2 causes only minimal physiological or developmental abnormalities, it is one of the most widely used chemical induction systems [99]. For example, the XVE-based system was used in transgenic *Arabidopsis* and tobacco plants for E2-inducible expression of the *GFP* reporter gene [99] and the *WUSCHELL* (*WUS*) target gene, which encodes a homeodomain protein involved in specifying stem cell fate in shoot and floral meristems and promoting the vegetative-to-embryogenic transition in transgenic *Arabidopsis* plants [100]. Using the XVE system, it was shown that the induced expression of both the *WUS* and *ONOPTEROS/AUXIN RESPONSE FACTOR 5*∆ (*ARF5*∆) genes lead to the activation of auxin signaling in *Arabidopsis* roots and enhancement of regeneration efficiency during shoot-induction stage of regeneration [101]. The XVE system was also tested in an important food crop *O*. *sativa*. It was shown that expression level of the *GFP* reporter gene is dependent on E2 concentration and constitutive expression levels of XVE in transgenic rice [102]. The developed XVE-based inducible MultiSite Gateway system for *Arabidopsis* provides high-throughput cloning of inducible constructs specific to a certain cell type in a single step, which guarantees numerous possibilities for creating inducible plant transgenic lines [103]. 

To allow constitutive transgene expression after chemical induction in rice, the XVE-controlled Cre/loxP-mediated recombination system was developed (Figure 6G) [104]. The chimeric transcription factor XVE was activated by soaking transgenic rice seeds in E2 solution, inducing expression of the Cre recombinase. Subsequent Cre/loxP-mediated recombination led to the fusion of constitutive maize ubiquitin-1 promoter with the *β-glucuronidase* reporter gene or the hpRNAi cassette targeted to a rice *phytoene desaturase* (*OsPDS*) gene. Constitutive and high expression of the target gene or the RNAi cassette remained in transgenic rice plants from induced germinating seeds. A similar XVE-controlled Cre/loxP-mediated recombination system was used earlier in transgenic tomato (*Solanum lycopersicum*) plants to induce constitutive high expression of the synthetic *B. thuringiensis* endotoxin gene *cryIAc*, conferring resistance to lepidopteran insects [105]. This system has also been optimized for use in transgenic rice by suppressing *OsSPS1*, a gene encoding sucrose phosphate synthase [106]. The efficiency of pOp6/LhGR and XVE systems was compared by hairpin RNA (hpRNA)-mediated gene silencing in transgenic *Medicago truncatula* roots [107]. The target genes contained hairpin cassettes to the *YFP* and *acetyl-CoA carboxylase* genes. It was shown that the YFP fluorescence was reduced in roots transformed with the components of the pOp6/LhGR system in the presence of dexamethasone, whereas the removal of the inducer did not reverse YFP inhibition. In roots transformed with the components of the XVE system, the YFP expression was low, even in the absence of E2. Since the leakiness of the XVE system in *Medicago* may be due to significant concentrations of phytoestrogens in legumes, only the dexamethasone-inducible system was chosen for silencing of the vital *acetyl-CoA carboxylase* gene in *Medicago* roots. As a result, prolific root growth was observed in transgenic *Medicago* plants until dexamethasone was applied and a significant reduction in *acetyl-CoA carboxylase* mRNA was detected in the presence of dexamethasone [107]. 

Additionally, a dual-controlled TGV system based on the combination of the dexamethasone-inducible tool with the Tet-dependent expression system (Figure 6H) was applied in transgenic tobacco plants [108]. The chimeric TGV protein consists of the tetR DBD, the rat GR LBD and the VP16 AD. In the absence of dexamethasone, TGV is inactive due to binding to the Hsp90 protein. Upon dexamethasone addition, TGV separates from Hsp90 and activates the expression of the *β-glucuronidase* gene driven by chimeric promoter containing seven repeats of the tetracycline operator (tetO7) sequence. Upon Tet addition, TGV protein dissociates from the reporter gene promoter and the level of the *β-glucuronidase* transcription goes to the uninduced level. 

Since application of Tet and steroids in large-scale field experiments is highly undesirable, insecticide-inducible expression tools were developed [109,110]. These tools are based on the chimeric GVEcR protein consisting of GR AD and DBD, the VP16 AD and worm ecdysone receptor (EcR) LBD. One variant of these systems uses a commercially available non-steroidal ecdysone agonist, tebufenozide, as an inducer [109] and the LBD of the EcR of the lepidopteran species *Heliothis virescens* (tobacco bud worm). In the absence of tebufenozide, the chimeric transcription factor GVEcR does not bind the glucocorticoid response element (GRE) in the promoter region of the *β-glucuronidase* reporter gene. The addition of tebufenozide activates GVEcR, which, in its turn, activates the *β-glucuronidase* gene expression in tobacco transgenic plants [109]. Another variant of the insecticide-inducible system based on a commercially available non-steroidal EcR agonist, methoxyfenozide, contains EcR LBD from the spruce budworm [110]. The chimeric transcription factor consists of the EcR LBD, the Gal4 or LexA DBD and the VP16 AD. The *luciferase* reporter gene was placed downstream of the Gal4 or LexA binding sites and minimal CaMV35S promoter (Figure 6I). Upon induction, the transcription activators induce expression of the *luciferase* reporter gene in transgenic *Arabidopsis* and tobacco plants. It was shown that the level of transgene expression was methoxyfenozide dose-dependent and that methoxyfenozide has an exceptional health and environmental safety profile [110]. However, it should be noted that not all chemical ligands always work successfully at different stages of plant development for inducible transgene expression experiments. For instance, *Arabidopsis* dormant seeds do not exhibit testa rupture and entry of some chemical ligands (i.e., methoxyfenozide) is limited. It has been shown that nitrate is the best permeable chemical ligand for *Arabidopsis* seed testa. When nitrate binds to the *NITRITE REDUCTASE 1* (*NIR1*) gene promoter, expression of the target gene is activated. This system is field applicable and can be used for seed germination recovery and enhancement technologies [111].

Chemical systems also include copper-inducible expression tools. Unlike the previously described artificial chemical inducers, copper is one of the essential microelements registered as a fungicide for field use in non-toxic concentrations. The copper-inducible transgene expression system consists of the yeast copper-regulated transcription factor ACE1 fused to the VP16 AD under the control of a constitutive promoter, and a reporter gene under the control of minimal promoter linked to the metal responsive element (MRE) (Figure 6J) [112]. Strong transcription activation of the *GFP* reporter gene was observed in transgenic *Arabidopsis* plants and in tobacco BY-2 cells in the presence of copper. Copper-induced activation of the *flowering locus T* (*FT*) reporter gene results in an early flowering phenotype with a flower bud on the top of transgenic *Arabidopsis* seedlings. Previously, a copper-inducible system was used in transgenic *Arabidopsis* plants to control expression of the *GFP* reporter [113]. The time-course of up- and down-regulation of the *GFP* expression in response to copper level, the optimal copper concentration and the tissues of *GFP* expression in three transgenic *Arabidopsis* lines were described [113].

Ethanol is also used to control transgene expression in plants. Earlier, it was shown that in the filamentous fungus *Aspergillus nidulans*, the transcription factor AlcR induces transcription of the *AlcA* gene upon ethanol addition [114]. The ethanol-inducible system consists of the transcription factor AlcR under the control of the constitutive viral promoter CaMV35S and a target gene driven by the chimeric promoter containing the CaMV35S minimal promoter fused at the TATA box to the upstream promoter sequences of the *AlcA* gene [115]. This system was used for ethanol-induced expression of the *β-glucuronidase*, *GFP* and *luciferase* reporter genes in transgenic *Arabidopsis* plants (Figure 6K) [115]. The ethanol-inducible system was combined with the Gal4/UAS system for the cell type-specific *AlcR* gene expression in *Arabidopsis* [116]. This combination was used for the spatially restricted ethanol-inducible expression of the *β-glucuronidase* and *GFP* reporter genes in transgenic *Arabidopsis* plants. Additionally, this system was used for induction of target gene expression in *C*. *reinhardtii* [117]. The ethanol-inducible system was also adjusted for application in monocotyledonous plants [118]. To achieve that, the chimeric promoter of the reporter gene was modified in comparison with the original system to increase ethanol inducibility. This system was tested in transgenic sugar cane for regulated expression of the *β-glucuronidase* gene by treating the roots of plants with ethanol. The advantages of this system are that it is suitable for field application and has a fast response. However, recently, it was shown that the *AlcR* gene alone leads to the retardation of growth in several tissues and several stages of growth in transgenic *Arabidopsis* plant lines in the presence of ethanol [119]. 

## 3. Discussion

The last three decades have witnessed the emergence of different systems for regulation of transgene expression in plants. One of the most important tasks of plant biotechnology was to find and characterize tissue- and cell type-specific promoters enabling stage-specific spatial control of the transgene expression. However, such promoters lack the ability to arbitrary control gene expression in time upon additional demands. Inducible systems help to overcome this limitation and allow to control timely transgene expression in particular tissues of interest without inhibiting plant regeneration. The chemical induction systems are the most widely used in plants. However, both optogenetic and chemical induction systems were successfully applied only in several model plants, including dicotyledonous plants *Arabidopsis*, tobacco, legume and monocotyledonous food crops (rice, maize, sugar cane).

Despite the fact that some of these inducible systems have a good potential to be applied in large-scale field experiments, so far, they have only been used in basic science (Table 1). Optogenetics has just started to be used in plants, and its main challenge, the unwanted transgene activation under ambient light, is yet to be addressed before its introduction to applied research. Light required for plant growth and development causes unwanted triggering of the optogenetic switches and constrains the transfer of this technology to applied research. Nevertheless, several approaches have recently been developed to overcome these limitations intrinsic to plants. For instance, in the PULSE system [67], the problem was addressed by combination of two optogenetic tools induced by far-red and blue light, respectively. As a result, the system is inactive in darkness or under ambient light and is induced only by far-red light. However, it should be noted that the PULSE system might still not be useful for field experiments. Namely, the PhyB and PIF6 proteins from *A. thaliana* may potentially interact with endogenous metabolic pathways of plants, affecting their physiology. Thus, a slight modification of the PULSE system seems to be necessary before it can be used in applied research. For instance, PhyB and PIF6 proteins could be replaced by those from the BphP1-QPAS1 system [120], which are activated by NIR light and, given their origin from bacteria *Rhodopseudomonas palustris*, should have no or minimal adverse effects on plant physiology.

Among chemically-inducible transgene expression systems, Tet and steroids are not suitable for applied research, since treating plants and soil with antibiotics or hormones will produce more harm that benefit. In comparison with these systems, the insecticide-inducible system has shown more potential to be applied in field experiments. However, insecticides should also be used with caution, since, for instance, methoxyfenozide is known to be slightly toxic to aquatic species and earthworms. It should be noted that insecticides may leach to groundwater and may be persistent in soil systems [121]. Copper-inducible system seems to have a high potential for application in field experiments (Table 1). Copper is not persistent in soil but can be stable in water; it is moderately toxic to most fauna and flora [121]. Therefore, copper may be used at low non-toxic concentrations in large-scale field experiments. Ethanol-inducible system has also shown good application prospects in field experiments. However, ethanol is unstable, and plants produce ethanol under hypoxia [122], which may cause unwanted transgene expression. 

Thus, currently, there is no ideal optogenetic or chemical induction system, which may be used in field experiments without caution or some limitations. At any rate, the choice of system type depends on the transgene of interest, the plant species and the specific application. Possibly, a combination of tissue-specific promoters with dual-controlled systems using light- and chemical induction will help to develop an ideal inducible system for plants. Of even greater significance is the development of multi-inducible systems for independent regulation of several target genes. In the future, the tools of this kind will allow one to protect food crops from adverse climatic and chemical conditions (drought, toxins, soil salinity, and radiation), plant diseases (molds, bacteria, and viruses), and pests (insects, and rodents). Potentially, these systems could also prevent the transfer of genes from transgenic to wild plants, reduce the consumption and accumulation of pesticides, poisons and fertilizers in plants. Further development of agricultural biotechnologies will allow for the creation of non-toxic and safe tools for high-quality food production.

## Figures and Tables

**Table 1:** Application of optogenetic and chemical induction systems in basic science and their potential use in applied research.

Inducible System	Use in Basic Science				Potential for Use in Applied Research and Notes	
	Organism	Purpose	Features	Ref		
Optogenetic induction systems						
System based on the PhyB-PIF6 interaction	*A. thaliana* *N. benthamiana*	Reversed far-red light-induced transcription activation of the target gene.	The PCB chromophore is produced in plant cells.	[60]	Low	PhyB is a plant phytochrome that can interact with the endogenous proteins of the plant cells, affecting endogenous metabolic pathways. This system can be unintentionally activated under ambient light.
System based on the CarH photoreceptor	*A. thaliana*	Green light-induced transcription inhibition of the target gene.	Plant photoreceptors show reduced activity in green light.	[61]	Low	The AdoB12 chromophore is not produced in plants. This system can be unintentionally activated under ambient light.
System based on the CRY2-CIB1 interaction	*C. reinhardtii*	Blue light-induced hydrogen production in transgenic alga.	The FAD chromophore is produced in all plant cells.	[66]	Low	This system can be unintentionally activated under ambient light.
PULSE system	*A. thaliana*, *N. benthamiana*	Dual-controlled system for transcription activation of the target gene.	The system is insensitive to ambient light.	[67]	Moderate	This system cannot be accidentally activated under ambient light. However, interaction of PhyB and PIF6 with endogenous proteins could affect plant physiology.
BLINK1 system	*A. thaliana*	Blue light-induced regulation of stomatal kinetics.	The FMN chromophore is produced in all plant cells. BLINK1 improves water use efficiency without penalty in carbon fixation.	[70]	Low	This system can be unintentionally activated under ambient light.
Chemical induction systems						
Tet-derepressible system	*N. benthamiana*	Tet-induced transcription derepression of the target gene.	Low background expression level, small amount of Tet to launch the system.	[79,81,82]	Low	Tet is an antibiotic and its usage in field experiments should be avoided.
Tet-off system	*A. thaliana*, *P. patens*, *N. benthamiana*	Tet-induced transcription inhibition of the target gene.	Low background expression level, small amount of Tet to launch the system.	[83,84,85]	Low	Tet is an antibiotic and its usage in field experiments should be avoided.
GVG/UAS system	*A. thaliana*, *N. benthamiana*, *L. japonicas*	Steroid-induced transcription activation of the target gene.	The system has low background expres-sion level.	[86,87,88]	Low	Dexamethasone is steroid and its usage in field experiments is highly undesirable. Steroid-inducible systems can cause severe growth disturbance in transgenic plants.
pOp6/LhGR system	*A. thaliana*, *N. benthamiana*, *O. sativa*, *Zea mays*	Steroid-induced transcription activation of the target gene.	Practically complete inhibition of the LhGR activity in the absence of inducer.	[89,90,91,92,93,94,95]	Low	Dexamethasone is steroid and its usage in field experiments is highly undesirable. Steroid-inducible systems can cause severe growth disturbance in transgenic plants.
β-estradiol (E2) induction system	*Zea mays*	E2-induced transcription activation of the target gene.	Low amount of inducer is needed to activate the system.	[96]	Low	Despite the fact that E2 causes minimal physiological or developmental abnormalities, the usage of steroid-inducible system in field experiments is highly undesirable.
XVE system	*A. thaliana*, *N. benthamiana*, *O. sativa*, *S. lycopersicum*, *M. truncatula*	E2-induced transcription activation of the target gene.	The system has low background expression level	[99,100,101,102,103,104,107]	Low	Despite the fact that E2 causes minimal physiological or developmental abnormalities, the usage of steroid-inducible system in field experiments is highly undesirable.
TGV system	*N. benthamiana*	Dual-controlled dexamethasone-induced transcription activation and Tet-induced transcription inhibition of the target gene.	The system has low background expression level.	[108]	Low	The usage of steroid dexamethasone and antibiotic Tet in field experiments is highly undesirable.
Insecticide-induced systems	*A. thaliana*, *N. benthamiana*	Insecticide-induced transcription activation of the target gene.	These systems have low background expression level.	[109,110,111]	Moderate	These systems can be applied in large-scale field experiments and can be used for seed germination recovery and enhancement technologies.
Copper-induced system	*A. thaliana*, *N. benthamiana*	Copper-induced transcription activation of the target gene.	Copper is already registered as a fungicide for field use in non-toxic concentrations.	[112,113]	High	This system is applicable in large-scale field experiments with copper used in low non-toxic concentrations.
Ethanol-induced system	*A. thaliana, Saccharum officinarum*, *C. reinhardtii*	Ethanol-induced transcription activation of the target gene.	The system has a fast response.	[115,116,118,119]	High	System is suitable for field application. However, plants produce ethanol under hypoxia that may cause unwanted transgene expression.
